# Fusion Method of Experiment and Finite Element for Constructing Process Performance Dataset of 22MnB5 Steel in Low-Temperature Hot Stamping

**DOI:** 10.3390/ma19173642

**Published:** 2026-08-27

**Authors:** Fangfang Li, Liang Wang, Run Wu

**Affiliations:** School of Intelligent Manufacturing and Control Engineering, Shanghai Polytechnic University, Shanghai 201209, China; 20251510083@sspu.edu.cn (L.W.); 20231510062@sspu.edu.cn (R.W.)

**Keywords:** 22MnB5, low-temperature hot stamping, dataset construction, springback angle, Vickers hardness

## Abstract

Performance prediction, process parameter optimization, and various data-driven research for low-temperature hot stamping (LTHS) processes all rely on abundant, continuous, and reliable process performance sample data. Collecting data merely through physical experiments leads to high costs, long test cycles, and limited coverage of working conditions. This paper focused on the LTHS process of 22MnB5 high-strength steel and proposed a dataset construction method that integrates experiments with finite element simulation. Firstly, LTHS experiments of 22MnB5 steel V-shaped parts were conducted under different combinations of forming temperature, in-die holding time, and stamping speed. Key performance parameters such as temperature field, forming springback angle, and Vickers hardness were measured. Secondly, a thermo-mechanical-phase transformation multi-field coupled finite element model (FEM) was established and validated using the experimental data. The results revealed that the simulation results agree well with the experimentally measured springback angle and Vickers hardness, and the FEM could accurately characterize the forming features and material property evolution throughout the whole LTHS process. On this basis, an experiment–simulation data integration framework was constructed: validated FEMs were adopted to supplement missing working conditions within the parameter space based on physical test samples. An LTHS integrated dataset with wide coverage, high data continuity, and strong usability was built by unifying variable definitions, sample organization modes, and standardized data formats. The dataset established in this paper can provide solid data support for the development of performance prediction models, process parameter optimization, and other data-driven studies of LTHS processes. Moreover, this dataset construction strategy integrating experiments and simulations can be extended to other metal plastic forming fields.

## 1. Introduction

With increasing demands from the automotive industry for lightweight body structures, crash safety, and low-carbon production, hot stamping technology has become a core manufacturing process for ultra-high-strength steel components in vehicles. 22MnB5 steel, owing to its excellent hardenability and formability, is one of the most widely used materials specifically designed for hot stamping applications. The conventional high-temperature hot stamping process requires heating the blank to 900–950 °C for complete austenitization followed by direct forming. This not only results in high energy consumption but also causes severe surface oxidation and decarburization of the sheet. Moreover, prolonged exposure to high-temperature loads significantly exacerbates thermal fatigue damage to the dies, constraining the green and low-cost development of the process. Mori et al. [1] reported that warm forming of 22MnB5 steel at approximately 600 °C resulted in a tensile strength of about 980 MPa, slightly lower than that obtained by conventional hot stamping, while the total elongation was significantly improved. Naderi et al. [2] further demonstrated that although the tensile strength of warm-formed parts was slightly lower than that of conventionally hot-stamped parts, their ductility and strength–ductility balance were significantly improved.

**Figure 1:**
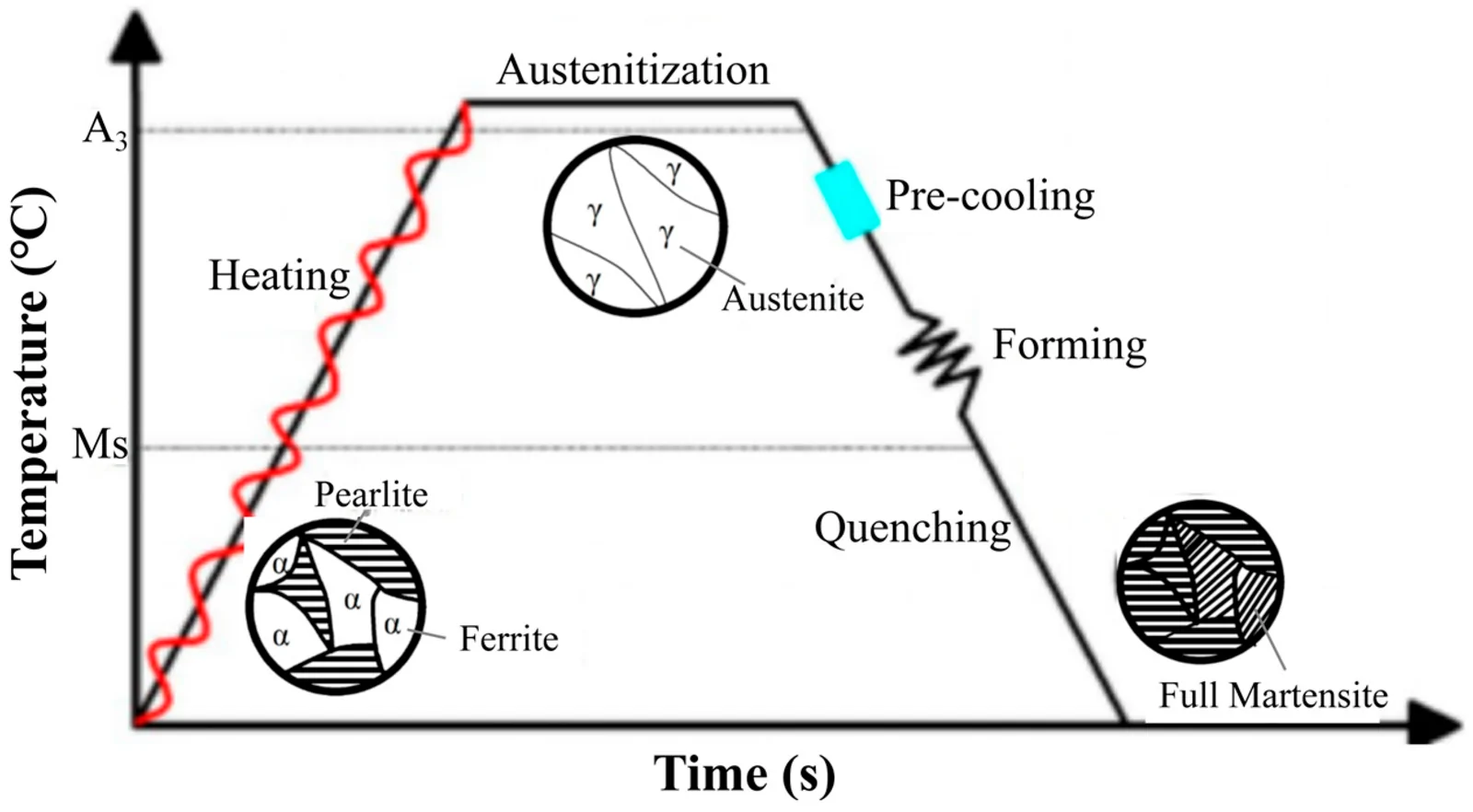
Process of LTHS.

LTHS emerges as a new generation of energy-saving hot forming technology. In this process, the austenitized blank is pre-cooled to a temperature above the martensite start temperature before stamping forming, and the technological route of LTHS is shown in Figure 1. Ganapathy et al. [3] reported that, for 22MnB5 steel, a pre-cooling temperature of approximately 500 °C provides a suitable forming window, which can reduce the flow stress and improve formability while ensuring sufficient phase transformation during subsequent die quenching. On the premise that the tensile strength of formed parts exceeds 1500 MPa, the in-die quenching time can be reduced by more than 60%. Meanwhile, thermal shock to dies and the oxidation degree of sheets are remarkably alleviated, endowing this process with multiple merits, including energy conservation, quality improvement, and cost reductions.

Balint et al. [4] proposed a process in which the blank was heated to the full austenitization temperature and then rapidly cooled to a temperature slightly above the martensite start temperature prior to forming. At this stage, the plastic deformation temperature was approximately 400 °C, where the material exhibits a relatively high strain hardening rate, and the forming limit was improved to a certain extent. Meanwhile, due to the reduced temperature, the in-die quenching time for each workpiece was significantly shortened. Ganapathy et al. [5] compared the performance of 22MnB5 steel between conventional hot stamping and forming after pre-cooling to 400 °C. They found that the yield strength of the low-temperature hot-stamped parts was approximately 1000 MPa and the tensile strength was approximately 1500 MPa, which were essentially comparable to those achieved by the conventional process. Moreover, due to more uniform cooling during forming, the hardness distribution of the parts become more consistent, and the overall mechanical properties exhibited better balance.

Despite the great potential demonstrated by low-temperature hot stamping in both theoretical research and some industrial practices, its widespread adoption remains constrained by multiple factors. Firstly, the lower forming temperature leads to reduced material flowability and restricted local strain, thereby increasing the risk of crack initiation. Secondly, under complex cooling paths, the microstructural evolution mechanisms have not yet been fully elucidated, often resulting in non-uniform local phase composition and distribution, which, in turn, causes performance fluctuations. In addition, residual stress and plastic springback are significantly enhanced after forming, imposing higher demands on the geometric accuracy control of the parts. These phenomena do not exist in isolation but originate from the deep coupling between thermodynamic processes and phase transformation behavior. It can be argued that the core challenge of LTHS lies in how to coordinate the deformation response and microstructural evolution of the material so that the two reach a balance in both time and space. To this end, systematic and in-depth research is still needed from the perspectives of material mechanism analysis, process parameter optimization, and process control strategies in order to achieve a fundamental understanding and controllability of this complex forming process. Meanwhile, the intertwining of microstructural evolution across temporal and spatial scales makes it difficult for traditional finite element methods, though capable of characterizing thermo-mechanical behavior and phase transformation laws to a certain extent, to fully reflect the complex reality of the LTHS process. The limitations are mainly manifested in three aspects: the large number of model parameters, high computational costs, and limited descriptive accuracy for nonlinear processes such as multi-phase transformation and stress relaxation. Especially in the low-temperature range, the thermo-mechanical response of the material exhibits stronger coupling and nonlinear characteristics, making traditional models often inadequate for predicting forming results or performing process optimization. At this point, in such a highly complex system, relying solely on analytical models can no longer meet the demands of efficient design and real-time control. Therefore, the introduction of flexible and efficient data-driven modeling approaches has become a necessary complement to traditional finite element analysis. Hart-Rawung et al. [6] developed a machine learning-based surrogate model for phase transformation kinetics in hot stamping, which could effectively replace complex phase transformation calculations and significantly reduce the overall computational time of hot stamping simulations. Murugesan et al. [7] employed support vector regression, decision trees, and random forests to predict the flow stress of steel under different strains, strain rates, and temperatures, demonstrating the capability of machine learning methods to characterize complex nonlinear hot-deformation behavior. Lejon et al. [8] applied machine learning-based anomaly detection methods to production-line process data for press hardening, enabling the identification of abnormal operating conditions and defective parts with high accuracy. By integrating experimental data and simulation results, it is possible to establish more adaptive and predictive process models, providing new technical support for the intelligent optimization and decision-making of low-temperature hot stamping.

Now, physical experimental data are authentic and reliable yet suffer from sparse samples and limited working conditions. Finite element simulation boasts powerful expansion capacity but relies entirely on experimental data for calibration and validation. Neither experiments nor simulations alone can meet the demands of big-data-driven research. Against this backdrop, this paper took the LTHS of V-shaped 22MnB5 steel components as the research object. A self-developed integrated experimental platform was constructed to acquire measured data, and a thermo-mechanical-phase transformation coupled finite element model (FEM) was established and validated. Furthermore, an experiment–simulation data fusion strategy was proposed. On the basis of measured experimental samples, the validated simulation samples were used to fill vacancies in the parameter space. A standardized integrated process performance dataset was established by unifying data formats and variable systems. The outcomes of this study can not only supply fundamental data support for property prediction and intelligent process optimization of LTHS, but also offer a referable technical paradigm for dataset construction in fields such as metal plastic forming and heat treatment.

## 2. LTHS Experiments of 22MnB5 Steel V-Shaped Parts

### 2.1. LTHS Experiment System and Scheme

The special automotive hot stamping boron–manganese steel 22MnB5 (supplied by BAOSTEEL, Shanghai, China) was selected as the test material in this research. The dimensions of the test specimens were as follows: total length of 100 mm, width of 60 mm, and sheet thickness of 1 mm. The as-received 22MnB5 steel exhibited a Vickers hardness of approximately 170 HV, a yield strength of approximately 400 MPa, a tensile strength of approximately 550 MPa, and an elongation of approximately 20%. The chemical composition of the 22MnB5 steel used in this study is summarized in Table 1.

**Figure 2:**
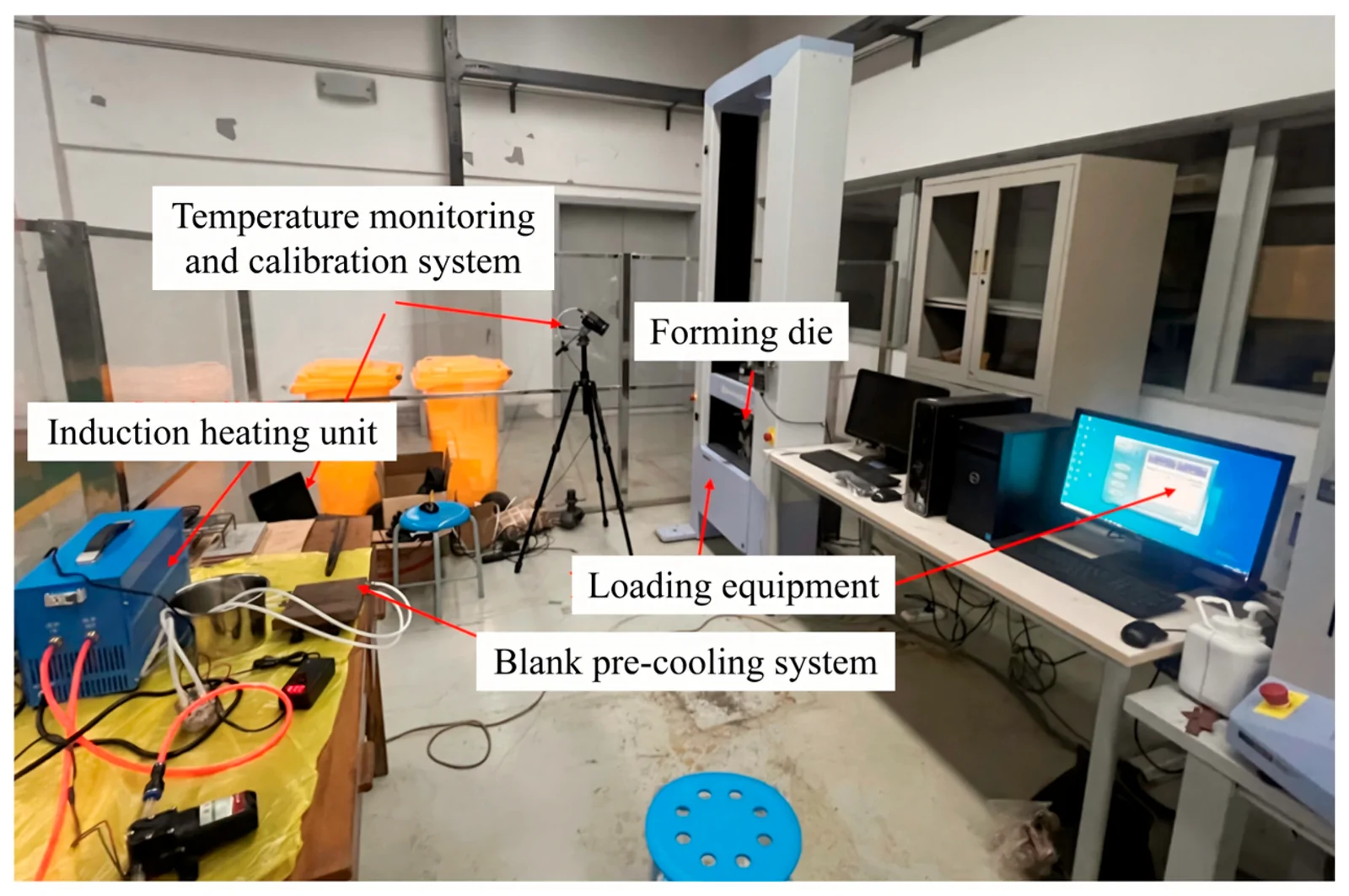
Experimental platform.

To realize full process control of the LTHS experiments, an integrated test platform integrating induction heating, precise pre-cooling, stamping forming, and full-field temperature monitoring was independently built, as presented in Figure 2. It can fully replicate the entire industrial LTHS process and realize high-precision control of blank temperatures, forming times, and heat transfer paths.

#### 2.1.1. Induction Heating Unit

The induction heating unit (School of Intelligent Manufacturing and Control Engineering, Shanghai Polytechnic University, Shanghai, China) adopts high-frequency induction heating equipment to rapidly austenitize blanks. The equipment has a rated maximum power of 5 kW and an operating frequency range of 10–100 kHz, which can heat 22MnB5 blanks to above 950 °C within a short period. The induction coil was designed with a spiral tower structure to ensure uniform magnetic field distribution and a consistent temperature across the plane and thickness of the blank. An infrared thermal imager was applied for real-time temperature measurement during tests. The measured results show that the maximum temperature difference in the core bending zone of specimens was less than 30 °C, satisfying the temperature uniformity requirements for LTHS experiments. The heating rate and target heating temperature of blanks can be precisely regulated by adjusting the number of coil turns and induction current.

#### 2.1.2. Blank Pre-Cooling System

**Figure 3:**
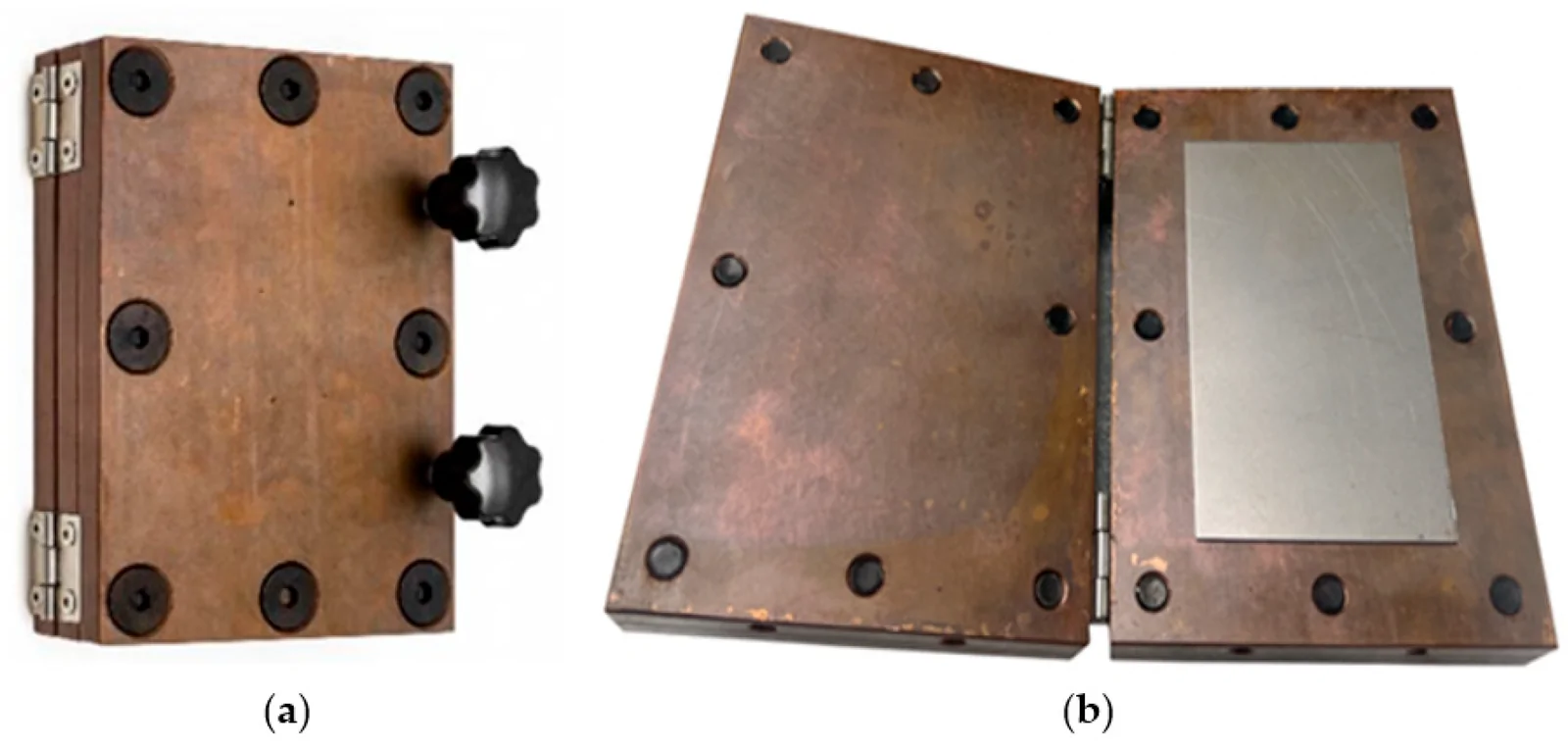
Blank pre-cooling system: (**a**) closed state; (**b**) open state.

The austenitized blank needs to be rapidly cooled to the target forming temperature range. In this study, a double-plate forced pre-cooling device (School of Intelligent Manufacturing and Control Engineering, Shanghai Polytechnic University, Shanghai, China) based on C18600 copper-chromium alloy was designed, as shown in Figure 3. This alloy possesses both high thermal conductivity and excellent thermal fatigue resistance. Optimized water channels were arranged inside the cooling plates, and the cooling intensity can be regulated by adjusting the cooling water flow rate and inlet temperature. The entire pre-cooling system can achieve an average cooling rate of over 40 °C/s with stable cooling performance. The cooling plate surfaces were precision-polished and coated with a corrosion-resistant nickel layer, which effectively reduces interfacial contact thermal resistance and extends the service life of the equipment.

After pre-cooling was completed, a dedicated fixture was used to transfer the specimen to the forming station within 3–5 s. The transfer duration and motion trajectory were strictly controlled to ensure that the forming start temperature drop was less than 30 °C, thereby guaranteeing test repeatability and temperature stability.

#### 2.1.3. Forming Die

**Figure 4:**
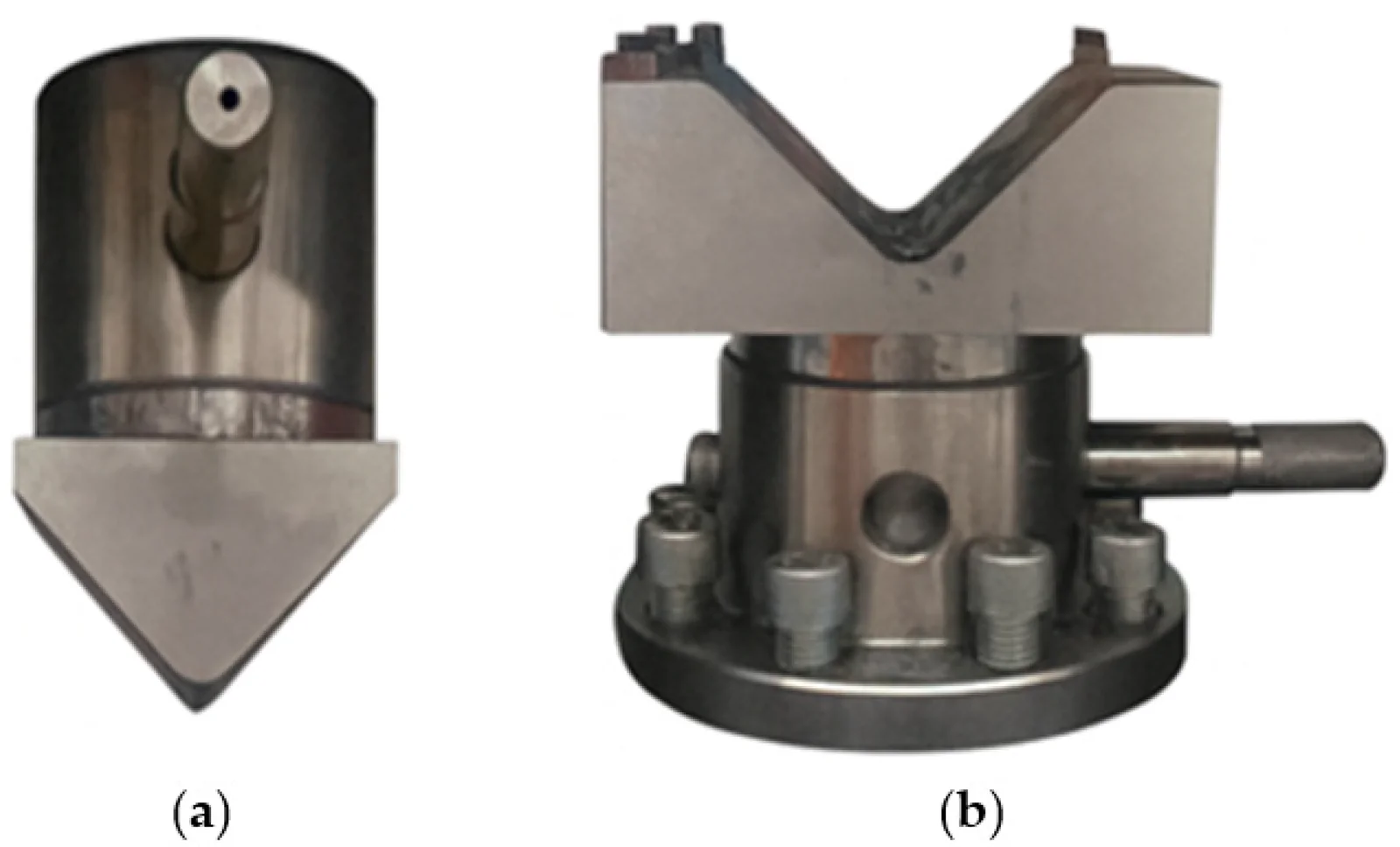
V-shaped forming die: (**a**) upper die; (**b**) lower die.

The stamping forming experiments were carried out on a Shimadzu universal testing machine (Shimadzu Corporation, Kyoto, Japan). The forming die (School of Intelligent Manufacturing and Control Engineering, Shanghai Polytechnic University, Shanghai, China) was made of H13 hot-work die steel, as shown in Figure 4, which exhibited high-temperature strength and thermal fatigue resistance. The die featured a 90° open V-shaped configuration, with the lower die rigidly fixed and the upper die driven unidirectionally downward by a hydraulic system. The stamping speed can be continuously adjusted within the range of 0–15 mm/s, with a displacement step accuracy of 0.01 mm, enabling forming experiments under different strain rates.

The die surface was mirror-polished to a surface roughness of Ra ≈ 0.8 μm to minimize the interference of interfacial friction on the forming results. Static loading calibration of the die was completed prior to the experiments, and the die temperature was maintained below 100 °C throughout the forming process, with temperature stability sustained by natural convection. After stamping, the upper die was held at the bottom dead center for 0–15 s to promote sufficient transformation from austenite to martensite. The loading system was equipped with an integrated force–displacement sensor and data acquisition module, enabling real-time acquisition of the forming mechanical curves, which provide a basis for the calibration of the finite element model.

#### 2.1.4. Temperature Monitoring and Calibration System

**Figure 5:**
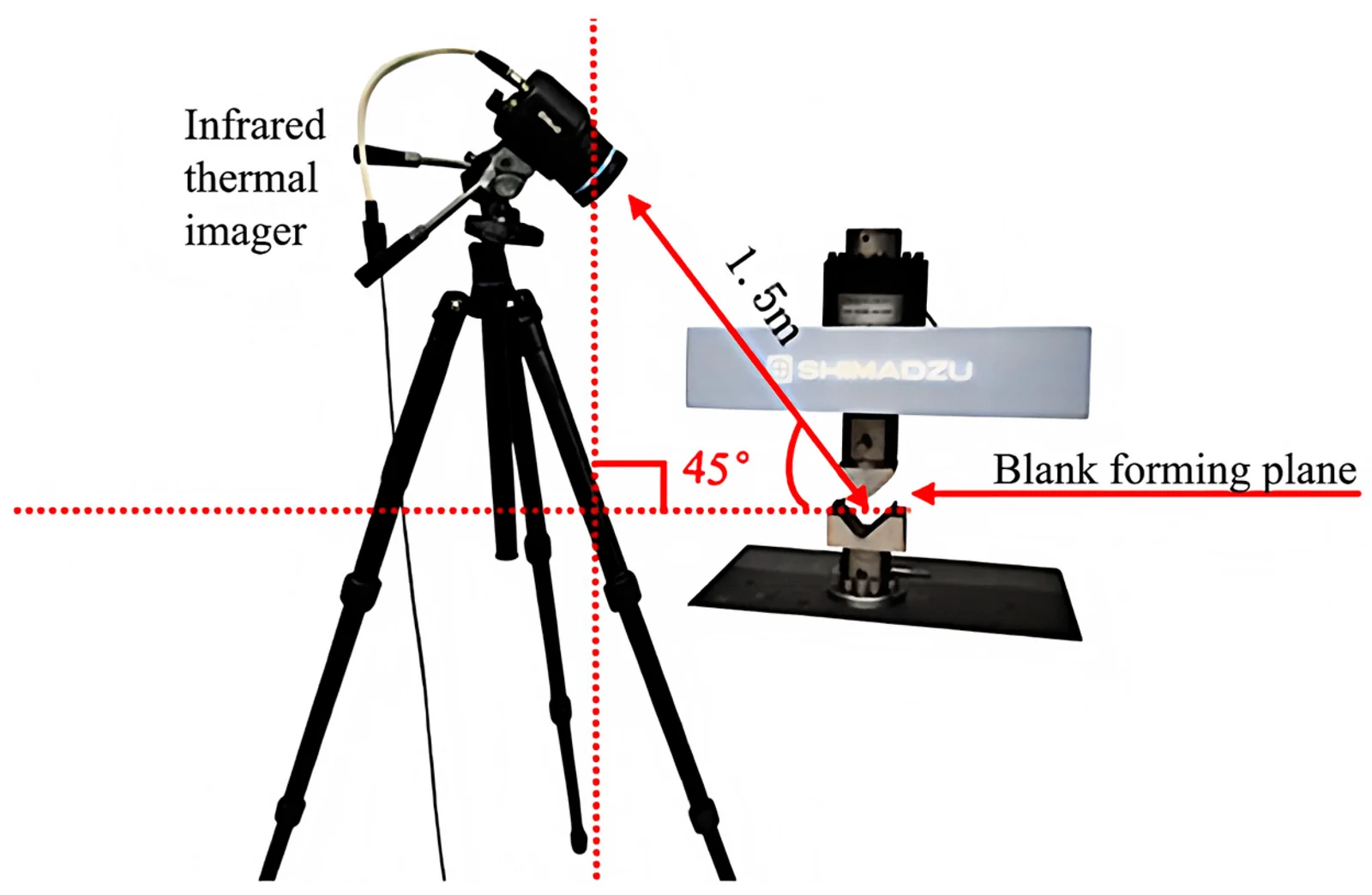
Thermal imager arrangement.

To achieve temperature field monitoring during the forming process, this study employed an FLIR infrared thermal imager (FLIR Systems, Inc., Wilsonville, OR, USA) for non-contact temperature measurements. The installation position and field of view of the thermal imager are schematically shown in Figure 5. Considering that the blank forming plane was fixed and the temperature range was concentrated in this study, the spatial arrangement of the thermal imager was precisely designed to ensure the accuracy and repeatability of thermal imaging measurements. The thermal imager was positioned at a straight-line distance of 1.5 m from the forming plane. This distance allowed full coverage of the entire V-shaped part region while avoiding thermal radiation saturation or field-of-view distortion caused by excessive proximity. Meanwhile, the optical axis was maintained at an angle of approximately 45° to the sheet surface, which effectively reduces the influence of specular reflection from the metal surface and minimizes temperature errors caused by high reflectivity. This arrangement ensured complete coverage of the V-shaped part forming area, preventing thermal radiation saturation and field-of-view distortion, and significantly mitigated temperature measurement errors induced by specular reflection from the metal surface.

### 2.2. Experimental Procedure and Sample Design

The specimens were first rapidly austenitized by induction heating, then transferred to the pre-cooling device and cooled to the target forming temperature range of 300–600 °C. Under a strictly controlled transfer time, the specimens were delivered to the forming station and stamped using a 90° V-shaped die, followed by holding at the bottom dead center for a certain period to promote subsequent phase transformation under the cooling effect of the die. After the holding stage, the specimens were cooled to room temperature, and then the springback angle and hardness of the formed parts were measured, thereby obtaining low-temperature hot stamping experimental samples under different process parameter conditions.

Considering that forming temperature, holding time, and stamping speed were the key control parameters affecting the forming behavior and performance response of 22MnB5 steel in LTHS, a Latin hypercube sampling method was adopted to design the experimental scheme to achieve comprehensive coverage of the process space and enhance sample representativeness. The target temperature range was 300–600 °C, holding time was 0–15 s, and stamping speed was 1–10 mm/s. A total of 200 process combinations were generated in the three-dimensional parameter space, with stratified sampling, minimum-distance screening, and boundary-weighted correction employed to balance central density. It should be noted that during the actual experiments, the temperature may deviate controllably from the target values due to factors such as heating batch variations, transfer time, and fixture heat exchange. Therefore, the measured temperature, rather than the preset temperature, was ultimately recorded in the data to ensure both process design consistency and data reliability.

Due to objective factors such as heating batch variations and heat exchange during transfer, the actual temperature of the specimens may deviate slightly but controllably from the preset values. Therefore, in this study, the measured temperature was uniformly adopted for database entry to further enhance data reliability. Each sample was assigned a unique identification number, with complete records of process parameters, measured temperatures, and performance indicators. The experimental parameter ranges and sample distribution characteristics are presented in Table 2.

## 3. Thermo-Mechanical-Phase Transformation Coupled FEM

The LTHS process was characterized by multi-physics coupling phenomena, such as plastic deformation, heat conduction, and solid-state phase transformation. Based on the Abaqus 2025 platform, a three-dimensional finite element model was developed in this study, which takes into account temperature-dependent material properties, interfacial contact conditions, friction behavior, and phase transformation kinetics.

### 3.1. Element Type Selection and Thermophysical Material Parameters

In finite element modeling, the selection of element types and modeling strategies for the sheet and the die have a significant impact on the accuracy of simulation results and computational stability. Previous studies have shown that when the forming temperature was in the range of 500–900 °C, the temperature difference between the surface and the center of the sheet can exceed 50 °C, and under cooling rates higher than 30 °C/s, the stress distribution along the thickness direction exhibits nonlinear characteristics [9]. If shell elements are adopted, it is often difficult to capture such temperature gradients and stress concentration phenomena. In contrast, solid elements can more realistically reflect failure modes such as thickness reduction, localized necking, and even crack initiation [10]. Therefore, in this study, the blank sheet was modeled using three-dimensional eight-node linear coupled thermo-mechanical compressible solid elements to more accurately characterize heat transfer and plastic deformation behavior along the thickness direction, thereby enhancing the physical fidelity and computational accuracy of the model under complex thermo-mechanical loads.

The thermophysical properties of 22MnB5 steel exhibit significant temperature dependence. The variation of density with temperature is negligible. However, the thermal conductivity gradually decreases with increasing temperature, indicating that the material’s heat dissipation capacity weakens at elevated temperatures. The specific heat capacity and coefficient of thermal expansion increase significantly with rising temperature, implying that the material more readily absorbs heat during high-temperature forming, thereby affecting heating efficiency and the cooling rate. Meanwhile, the coefficient of thermal expansion gradually increases with temperature. The aforementioned parameters directly affect heat transfer efficiency, thermal stress distribution, and die-matching clearance. In the modeling process, all these parameters were assigned using temperature-dependent fitting formulas. Additionally, boundary parameters such as the convective heat transfer coefficient and the interfacial contact heat transfer coefficient were defined. The specific thermophysical properties and heat transfer parameter fitting formulas were determined with reference to existing literature, as shown in Table 3.

The die was modeled as a rigid body with heat transfer capabilities. Since the die stiffness was much higher than that of the sheet, its elastic deformation was less than 0.1% of the blank sheet thickness, and thus it can be treated as a rigid body without the need for solid element discretization [15]. To accurately describe the contact and heat transfer characteristics during forming, a curvature-continuous transition structure was designed at the die radius, and mesh refinement was applied in the key contact regions to enhance geometric and heat flux resolution. The die surface temperature was set to 25 °C. Considering the significant influence of pressure on the heat transfer coefficient during the forming process, the heat transfer coefficients shown in Table 2 and Equation (1) were adopted as functions of both temperature and pressure to characterize the thermal conductivity of the material [16].

(1) $$h_{c}=h_{0}+\alpha p$$

where, $h_{c}$ is the actual heat transfer coefficient, $h_{0}$ is the initial heat transfer coefficient, α is the pressure sensitivity coefficient, and *p* is the contact pressure.

### 3.2. Contact, Friction, and Mechanical Boundary Conditions

In the FEM, the normal contact between the blank sheet and the die was modeled using the penalty contact algorithm. This method approximates the non-penetration constraint by introducing a virtual penalty stiffness at the contact interface. When the sheet comes into contact with the die and slight penetration occurs, the normal contact force can be calculated as Equation (2).

(2) $$F_{n}=k_{p}\delta_{n}$$

In the tangential direction, the Coulomb friction model was adopted, with a friction coefficient *μ* of 0.15 under hot stamping conditions, to reflect the friction enhancement effect caused by insufficient lubrication and oxide film rupture at elevated temperatures. Compared with the Lagrange multiplier method, the penalty method exhibits better numerical stability and computational efficiency in explicit dynamic solutions, and can naturally handle the frequent contact, separation, and sliding behavior between the sheet and the die during forming, while avoiding contact stiffening and oscillation issues. For LTHS simulations, this method ensured convergence while accurately capturing interfacial friction and heat transfer effects, providing reliable numerical support for stress distribution, springback prediction, and phase transformation kinetics analysis.

**Figure 6:**
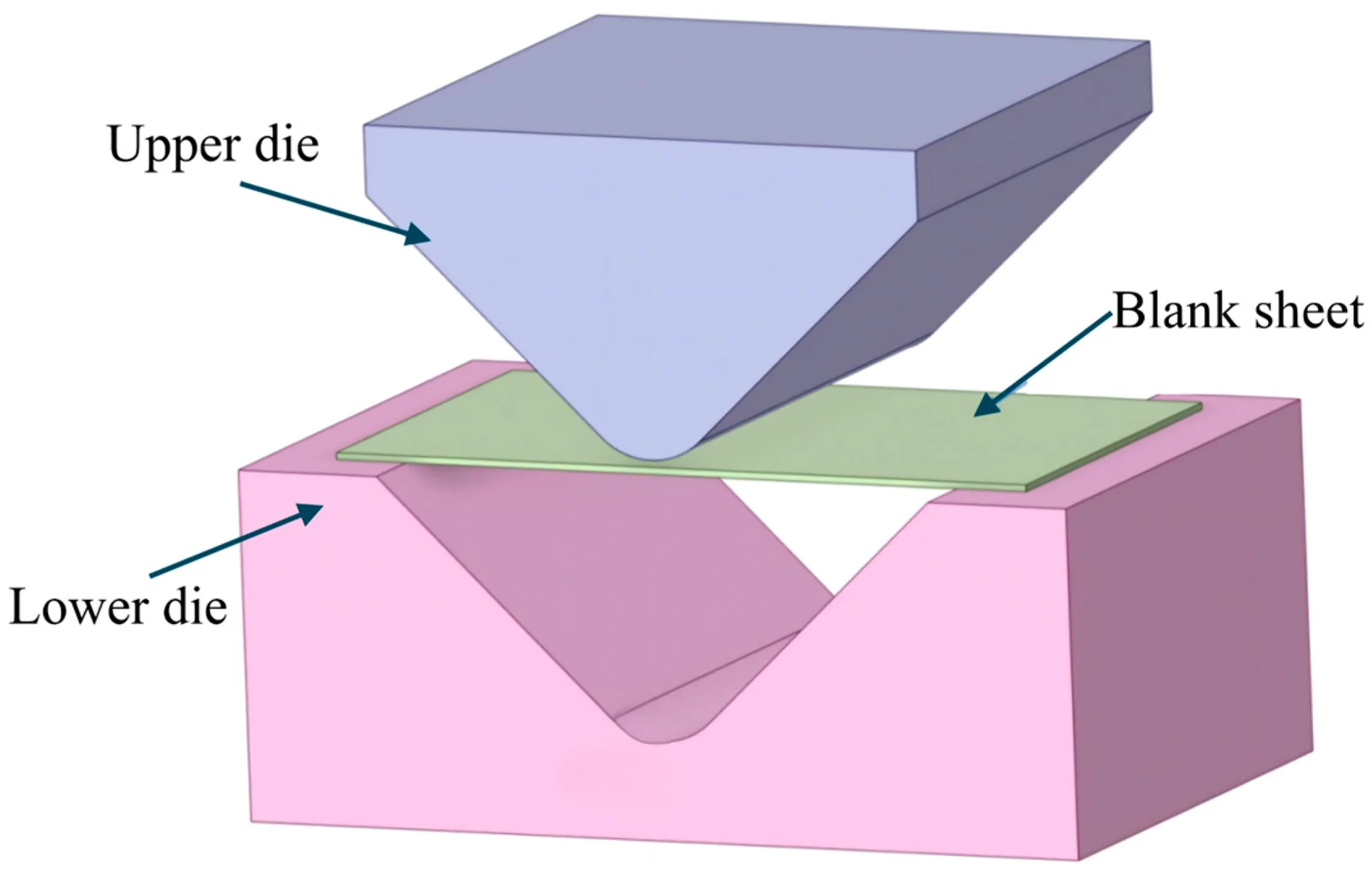
FE Modeling of LTHS of 22MnB5 steel V-shaped part.

Regarding mechanical boundary conditions, they mainly included the constraints and motion control of the upper and lower dies. The lower die remained completely fixed throughout the forming process, while the upper die was allowed to move only in the vertical direction with a single degree of freedom. The motion process was divided into three stages. The first was the loading stage, during which the upper die moved downward at the preset stamping speed, causing plastic deformation of the sheet. This was followed by the holding stage, during which the blank sheet was in full contact with the die, ensuring not only the geometric accuracy of the formed part but also promoting rapid heat conduction and microstructural transformation. Finally, in the unloading stage, the upper die was lifted, releasing the constraints and allowing the part to spring back. Based on the above modeling approach, the established finite element model for LTHS of 22MnB5 steel V-shaped parts is shown in Figure 6.

### 3.3. Automated Calculation Module for Phase Transformation and Springback

To achieve a more comprehensive simulation analysis of the LTHS process, this study relied on the phase transformation analysis module of Abaqus, combined with the USDFLD user subroutine and Python 3.14.7 secondary development plugins, to realize the integrated automation of phase transformation evolution and springback angle calculations, as well as post-processing. The overall implementation approach was similar to the previous J-integral-based related modules, all of which take the TTT diagram as the core input and track the microstructural evolution through the coupling of temperature history and phase transformation kinetics. The USDFLD subroutine was used for updating and transferring field variables during the solution process, while the Python side was responsible for parameter input, result reading, phase-fraction visualization, and automatic identification of the springback angle, thereby achieving integrated processing of phase transformation analysis and geometric result extraction, and improving the automation level and consistency of simulation post-processing. This method relied on the system’s native phase transformation solution capability and achieves integrated processing of data reading, phase-fraction visualization, and geometric angle identification through the plugin interface, significantly enhancing the efficiency and consistency of simulation post-processing.

In the phase transformation analysis part, the plugin called the built-in phase transformation solver of Abaqus and imported the TTT diagram data of 22MnB5 steel to perform real-time coupled calculation of the sheet temperature field and phase transformation kinetics. The diffusional transformation was described by the JMAK equation as shown in Equation (3). The martensitic transformation was represented by the Koistinen–Marburger equation given in Equation (4).

(3) $$\xi \left(t\right)=1-exp\left[-k(T)t^{n}\right]$$

(4) $$\xi_{M}=1-exp\left[-exp(K(M_{s}-T)\right]$$

The JMAK and Koistinen–Marburger models were adopted in this study because they provide a computationally efficient description of the two dominant types of phase transformation involved in the cooling of 22MnB5 steel and can be readily parameterized using the available TTT data. The JMAK model was used to describe time-dependent diffusional transformations, whereas the Koistinen–Marburger model was employed for the essentially athermal martensitic transformation below the martensite start temperature. This simplified formulation is suitable for the present study because the finite element model is primarily intended to describe the influence of phase transformation behavior on the macroscopic thermo-mechanical response and forming performance. Its applicability is therefore mainly restricted to thermal histories and process conditions covered by, or close to, the calibrated 22MnB5 TTT data. It should be noted that the present formulation does not explicitly account for transformation plasticity, latent heat associated with phase transformation, or phase-dependent constitutive flow behavior. Consequently, although it can reproduce the overall phase-fraction evolution and support the prediction of springback and hardness trends, its accuracy may be limited for detailed predictions of local stress evolution, transient temperature variations, and microstructural heterogeneity, particularly outside the calibrated process range.

By reading the temperature history of each element, the system can automatically determine whether it has entered the phase transformation range and generate phase distribution results. The simulation results indicated that the bending corner region, due to its close contact with the die and higher cooling rate, first undergoes the transformation from austenite to martensite, ultimately forming a microstructure dominated by hard phases. In contrast, the middle and edge regions of the unformed surface exhibited slower cooling, retaining a portion of soft phases, resulting in an obvious spatial stratification characteristic in the microstructure. Overall, the phase distribution was consistent with the hardness variation observed in the experiments, indicating that the built-in phase transformation model can accurately reflect the microstructural evolution trends in different regions.

In the springback angle analysis part, the plugin automatically extracted the nodal coordinates after forming and after unloading through the Abaqus post-processing interface, and calculated the opening angle of the V-shaped part using the geometric centerline as a reference. This method did not require manual calibration or post-measurement and can quickly obtain angle variation results under batch conditions. The simulation results showed that under low-temperature forming conditions, the springback trend was relatively more pronounced, while under high-temperature forming conditions, the plastic recovery of the material dominated and the geometric deviation was relatively small. The variation pattern of the springback angle with process parameters was consistent with the experimental measurement results, demonstrating the reliability of the model in predicting shape recovery.

## 4. Results Analysis and Fusion Dataset Construction

### 4.1. Experimental Results Analysis

Based on the Latin hypercube sampling scheme, a total of 200 valid LTHS experimental samples were obtained. To visually demonstrate the correspondence between process parameters and performance indicators in the experimental database, data from several typical working conditions are listed in Table 4.

As shown in Table 4, the forming temperature has a significant effect on the springback angle and Vickers hardness of 22MnB5 steel after LTHS. In the low-temperature range (approximately 300–370 °C), the specimens generally exhibited relatively large springback angles and lower hardness values. The springback angles of E1 and E2 reached 1.56° and 1.25°, respectively, while the hardness values were only 379.3 HV and 392.5 HV. As the forming temperature increased to the medium-temperature range (approximately 420–500 °C), the springback angle decreased significantly, and the Vickers hardness increased substantially, indicating that the material exhibited good forming compatibility and performance stability within this temperature range. When the temperature was further raised above 560 °C, the springback angle continued to decrease, and a negative springback phenomenon occurred under the 600 °C condition, suggesting that the geometric recovery behavior of the part undergoes a notable change at elevated temperatures. Overall, within the parameter range of this study, increasing the forming temperature was beneficial for reducing the springback angle and improving hardness. The comprehensive performance of the specimens was relatively stable in the 400–500 °C range, which can serve as an important reference interval for subsequent finite element validation and the construction of the experiment–simulation fusion dataset.

### 4.2. Validation of the Finite Element Model

The accuracy of the finite element simulation results directly determined the quality of subsequent dataset construction and the effectiveness of model training. To ensure that the numerical simulation can characterize the actual forming response throughout the entire LTHS process, this study established an equivalent finite element benchmark model based on the experimental conditions. Boundary conditions and load paths consistent with the experiments were set for each stage, including heating, transfer, forming, and in-die cooling. Systematic calibration was performed on the material constitutive model, interfacial friction, contact heat transfer coefficient, and die cooling parameters to achieve accurate mapping and consistent matching of process parameters. On this basis, the simulation results were compared and validated against the measured data. Quantitative validation was primarily conducted using the springback angle and Vickers hardness, while the temperature field evolution and phase-transformation results were used to assess the overall physical consistency of the simulation, to identify the sources of deviation and evaluate the robustness and applicability of the model, thereby providing reliable numerical support and engineering foundation for subsequent large-scale sample generation based on simulation data and the construction of multi-task learning models.

**Figure 7:**
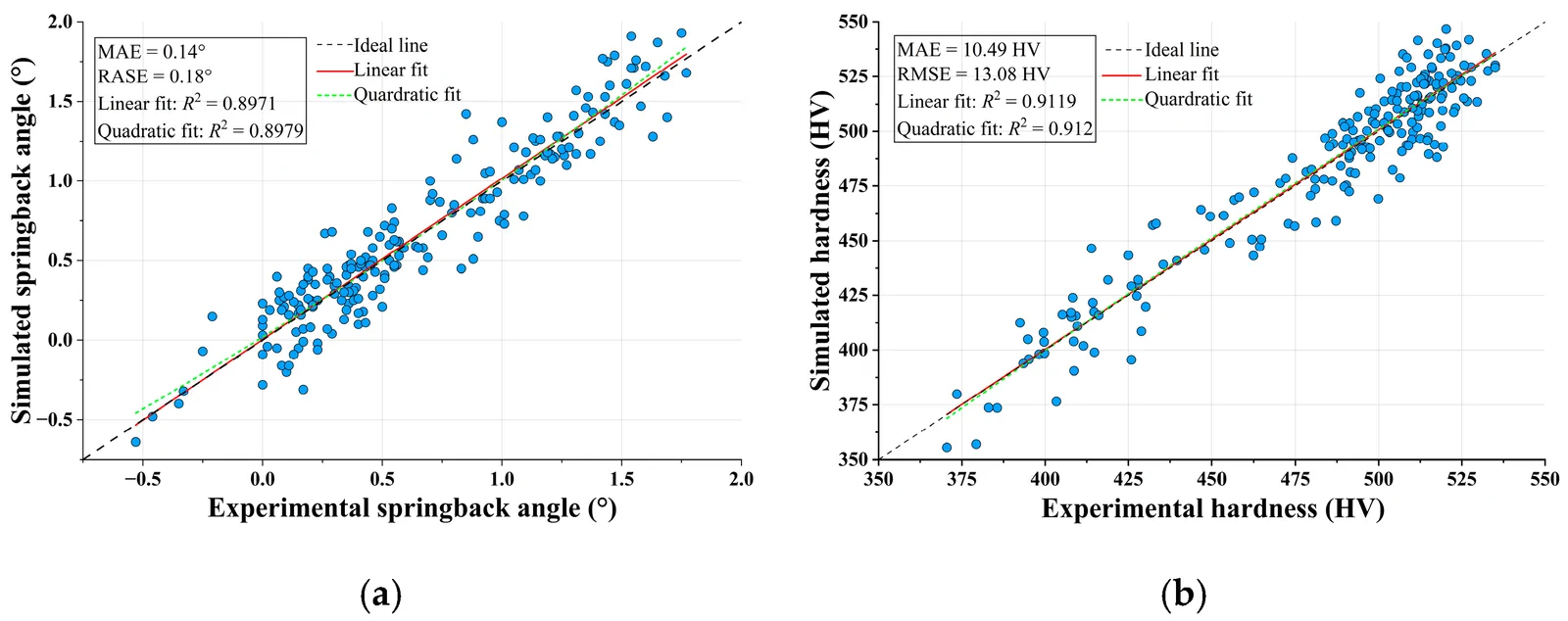
Comparison of FE and experimental results of springback and hardness: (**a**) springback angle; (**b**) hardness.

Figure 7 compares the experimental and simulated values of the springback angle and hardness. It can be observed that the two exhibit a highly consistent overall distribution trend. The data points were densely distributed on both sides of the ideal diagonal line, indicating that the simulation results were in good agreement with the experimental results in both magnitude and variation trends. The scatter points for both springback angle and hardness show a clear linear characteristic, with the fitting slope close to unity, suggesting high prediction accuracy of the model without significant systematic bias. To further evaluate the fitting characteristics in Figure 7, both linear and quadratic regressions were performed for the springback angle and Vickers hardness. For the springback angle in Figure 7a, the coefficients of determination (R^2^) obtained from the linear and quadratic regressions were 0.8971 and 0.8979, respectively. For the hardness results in Figure 7b, the corresponding R^2^ values were 0.9119 and 0.9120, respectively. Although the quadratic regressions yielded slightly higher R^2^ values, the increases were only approximately 0.0008 for the springback angle and 0.0001 for hardness. These negligible improvements indicate that introducing the quadratic term does not meaningfully improve the fitting accuracy. Therefore, considering the comparable fitting performance and the simpler model form, linear regression is more appropriate for describing the relationships between the experimental and simulated results. Although slight scatter exists for a few data points under low-temperature and short-holding-time conditions, the overall deviation remained limited. The maximum deviation of the springback angle was approximately 1.5°, and the hardness error was mainly within ±15 HV, both falling within the allowable fluctuation range of the forming experiments. The differences mainly arose from the treatment discrepancies of the boundary conditions between experiments and simulations, such as the actual thermal contact state between the die and the sheet, friction conditions, and the phase transformation rate differences of the material in the austenite-to-martensite transformation range. These factors were simplified in an averaged form in the model, leading to local deviations. However, the overall error was relatively small, verifying that the simulation results can truly reflect the actual process within a controllable range.

**Figure 8:**
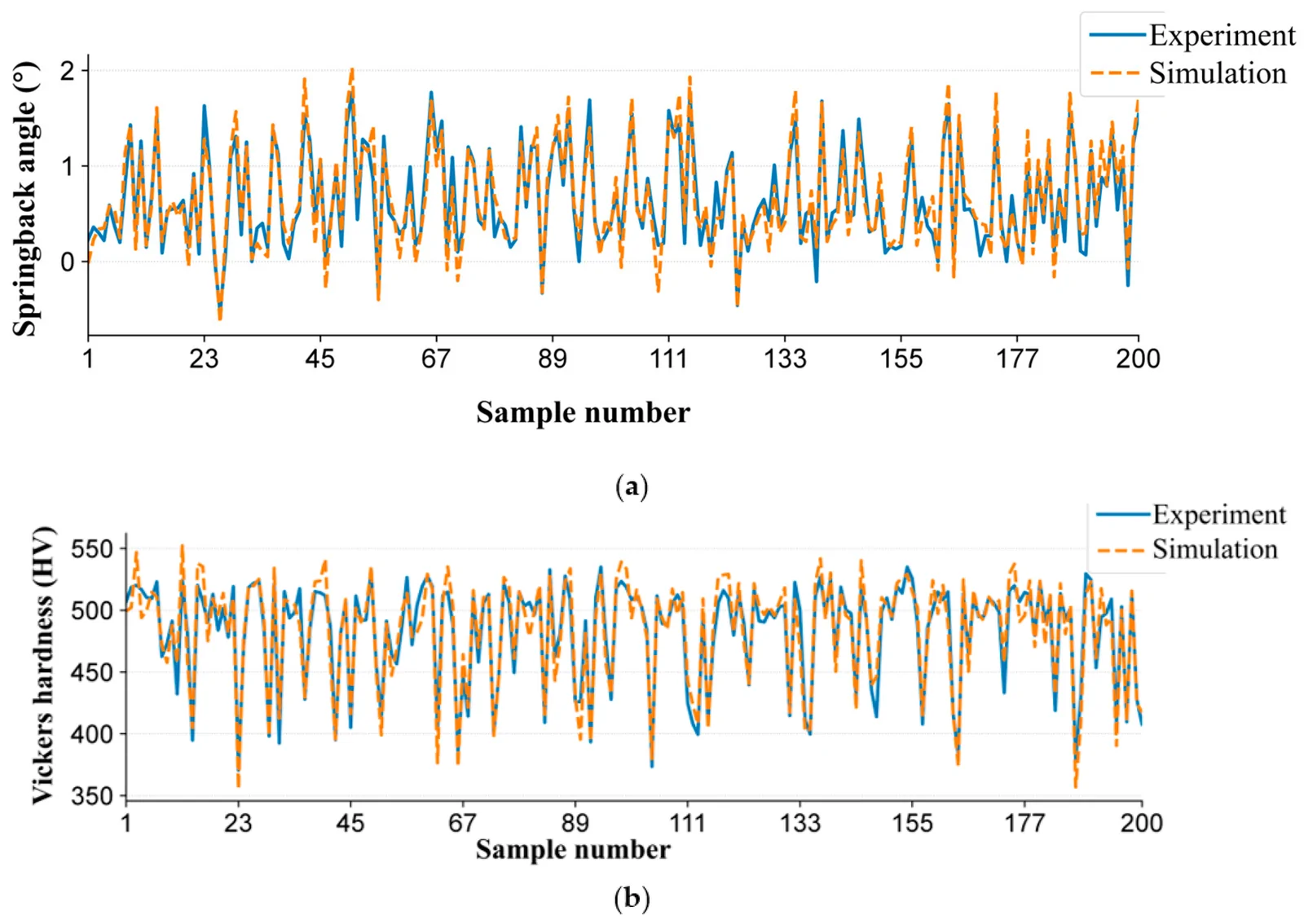
Comparison of experimental and simulated results: (**a**) springback angle; (**b**) hardness.

Figure 8 presents the overall distribution trends of the experimental and simulation results across 200 sample sets. As can be seen from this figure, the fluctuation patterns and magnitudes of the two datasets are highly similar, with the peak and valley positions essentially corresponding to each other, indicating that the variation trends under different process combinations are consistent between the two. In the springback angle curves, the simulation results are slightly lower than the experimental values, suggesting that the stress release in the simulation model during the elastic recovery stage was somewhat conservative. For the hardness curves, a slight systematic underestimation was observed, mainly attributable to the model’s incomplete consideration of the latent heat of phase transformation and the differences in actual die thermal conductivity. These discrepancies remain within a stable and acceptable range and do not affect the judgment of the overall trends. More importantly, the simulation and experimental results exhibited consistent distribution trends, with both showing the same variation patterns in terms of local fluctuations and overall tendencies. This indicated that although the simulation model involves certain idealized assumptions, it did not alter the direction of variation or the sensitivity characteristics of the key performance indicators.

**Figure 9:**
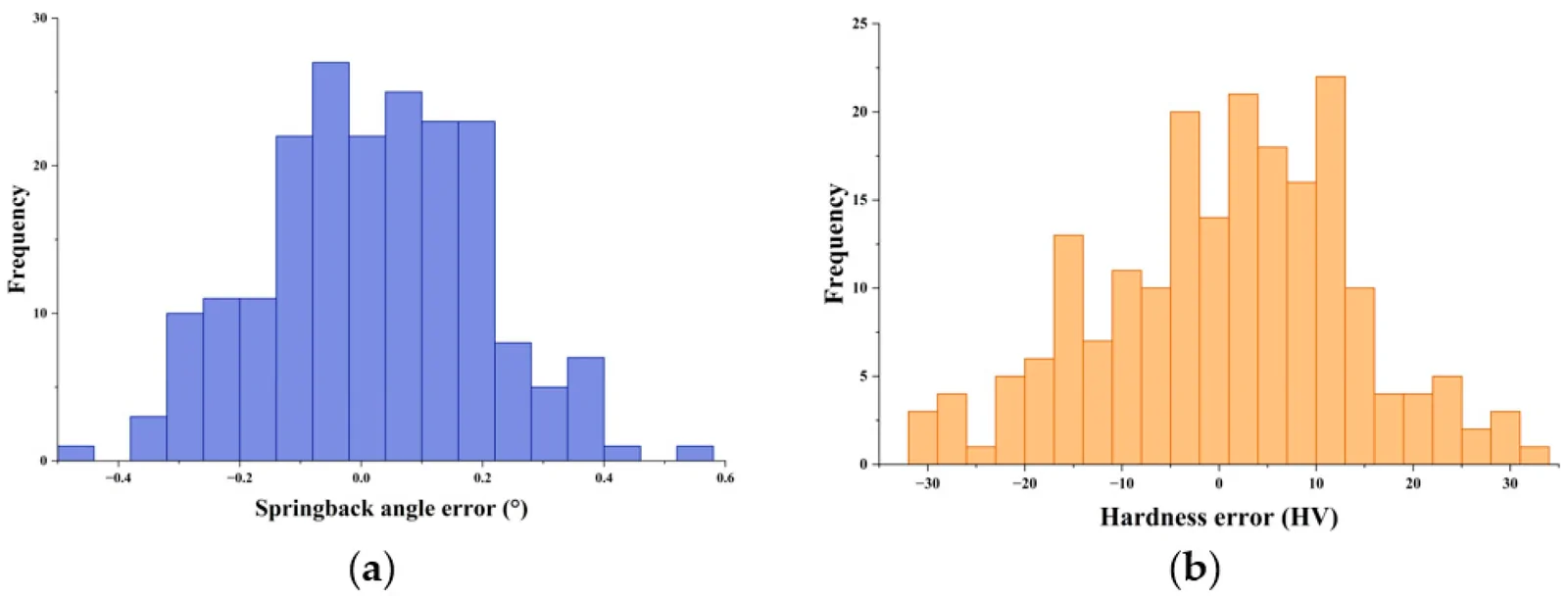
Comparison of error distributions between FE and experimental results: (**a**) springback angle; (**b**) hardness.

Figure 9 presents the error distributions of the springback angle and Vickers hardness, where the error is defined as the difference between the FE prediction and the corresponding experimental value under the same process condition. Both error distributions are centered around zero and show no obvious systematic shift. The springback angle errors are mainly concentrated within approximately ±0.5°, while most hardness errors are distributed within approximately ±15 HV, with only a small number of samples showing larger deviations. These results indicate that the discrepancies between the FE predictions and experimental measurements are mainly associated with random fluctuations rather than systematic model bias.

The discrepancies between simulation and experiment in terms of key performance indicators such as the springback angle and hardness were generally small, with clear sources of deviation and reasonable variation patterns. The simulation results can accurately reproduce the overall distribution characteristics and variation trends of the experimental data, with the deviation magnitude falling within the acceptable error range of the LTHS process. Quantitative comparison showed that the coefficients of determination between the simulated and experimental springback angle exceeded 0.89, and that for hardness prediction exceeded 0.91, indicating that the model exhibited high consistency and stability in both geometric accuracy and performance response. This demonstrated that the established finite element model can effectively describe the multi-physics coupling behaviors involving thermal, mechanical, and phase transformation aspects during the LTHS process. Although the model incorporated moderate simplifications in terms of thermal contact conditions and phase transformation kinetics, their effects were mainly manifested as local deviations with limited influence on the overall variation patterns. The validation results confirmed that the FEM can capture the forming characteristics and performance evolution laws in the actual process, exhibiting good engineering applicability. On this basis, the validated simulation results can be further used to supplement data samples for working conditions not covered by experiments, providing a reliable foundation for constructing the experiment–simulation fusion dataset.

### 4.3. Construction of the Experiment–Simulation Fusion Dataset

#### 4.3.1. Variable Definition and Sample Organization of the Dataset

To ensure that the experimental and simulation samples can be integrated and invoked within a unified framework, the input and output variables of the fusion dataset were first screened and defined. The variable selection followed three principles: first, variable homogeneity, meaning that the variables should be obtainable in both experiments and finite element simulations to ensure a consistent data structure for samples from different sources; second, strong correlation, meaning that the variables should have a major influence on the forming quality and performance response of LTHS; and third, dimensionality reduction, meaning that the number of variables should be moderate to avoid introducing too many weakly correlated variables when the sample size was limited, which could compromise the stability of subsequent data modeling. Based on the above principles, forming temperature, holding time, and stamping speed were selected as the input variables, while the springback angle and Vickers hardness were selected as the output variables. The variable composition of the dataset is presented in Table 5. Additionally, a sample source identification field was added to distinguish between experimental and simulation samples, ensuring data traceability.

The springback angle was used to characterize the geometric recovery of the part after unloading and served as an important indicator for evaluating forming accuracy. Vickers hardness reflected the mechanical performance level after forming and cooling, and was closely related to the microstructural transformation results. Although other process data, such as temperature fields and force–displacement curves, can also be obtained during the experiments, such data were primarily process monitoring information with relatively high dimensionality and were not directly suitable as target outputs of the process performance dataset at this stage. Therefore, in this study, they were mainly used for experimental process control and FEM validation, while the springback angle and Vickers hardness were adopted as the primary performance response indicators of the fusion dataset.

#### 4.3.2. Data Fusion and Pre-Processing Methods

After completing the experimental data collection and validation of the finite element simulation results, the experimental and simulation samples were organized and fused. First, the experimental samples were screened, and the data under each working condition, including the measured forming temperature, holding time, stamping speed, springback angle, and Vickers hardness, were extracted. Invalid samples caused by abnormal temperature acquisition, evident instability of the formed part, or missing hardness measurements during the experiments were excluded from the fusion dataset.

Second, the finite element simulation samples were screened. Considering that the simulation data were mainly used to supplement the working conditions not covered by experiments, only the simulation samples that had been validated against experimental results and exhibited errors within the acceptable range were selected for inclusion in the fusion dataset. The input variables of the simulation samples were consistent with those of the experimental samples, and the output variables, including the springback angle and Vickers hardness, were obtained from the simulation post-processing results.

Subsequently, the experimental samples and simulation samples were uniformly numbered and labeled with source identifiers. The experimental samples were numbered as E1, E2, ..., and the simulation samples as S1, S2, ..., to distinguish between experimental and simulation data. Each fused sample contained fields such as the sample number, data source, forming temperature, holding time, stamping speed, springback angle, and Vickers hardness.

Finally, the screened experimental samples and simulation samples were merged according to the unified field structure to form the experiment–simulation fusion data table. This data table preserved the original process parameters and performance response data, ensuring data traceability and providing fundamental data support for subsequent performance prediction, process parameter optimization, and data-driven analyses.

After organizing the experimental samples, expanding them via simulation samples, and unifying data organization, the experiment–simulation fusion dataset was finally established. Compared with the experimental data alone, this dataset covers a broader range in the process parameter space and exhibits a more continuous sample distribution, enabling a more comprehensive reflection of the combined effects of forming temperature, holding time, and stamping speed on the springback angle and hardness. Particularly in the process boundary regions that are difficult to extensively cover by experiments, the introduction of validated simulation samples effectively compensates for the deficiencies in both the quantity and distribution of experimental data.

From the perspective of dataset construction results, the experimental samples ensured the authenticity and representativeness of data within the key process ranges, while the simulation samples enhanced the extensibility of the dataset in the multi-parameter process space. The fusion dataset, formed by combining the two, not only preserves the direct characterization of actual process responses from experimental data but also leverages the advantages of finite element simulation in sample supplementation, endowing it with good engineering applicability and value for subsequent utilization. This dataset can serve not only as a foundation for analyzing the process laws of low-temperature hot stamping but also as a unified data support platform for subsequent development of performance prediction models, process parameter optimization, and other data-driven studies.

## 5. Conclusions

This study focused on the LTHS process of 22MnB5 steel, with research conducted around experimental data acquisition, finite element simulation validation, and the construction of an experiment–simulation fusion dataset.

1. An integrated LTHS experimental platform was independently built. Based on Latin hypercube sampling, 200 stamping experiments of 22MnB5 steel V-shaped parts were completed. The effects of three key parameters, forming temperature, holding time, and stamping speed, on the springback angle and Vickers hardness of the formed parts were clarified.
2. A thermo-mechanical-phase transformation multi-field coupled finite element model was established, combined with Python secondary development to achieve automated computation of phase transformation and springback. The model validation results showed that the coefficients of determination (R^2^) between the simulated and experimental results were 0.8971 for the springback angle and 0.9119 for Vickers hardness, demonstrating good agreement between the finite element predictions and experimental measurements, and showing that the model can accurately characterize the forming and performance evolution behavior throughout the entire low-temperature hot stamping process.
3. A dataset construction method integrating experiments and finite element simulations was proposed. Based on measured experimental data and supplemented by validated simulation data, a standardized fusion dataset was constructed with unified data formats and variable systems. This dataset features wide sample coverage, strong continuity, and good traceability, providing data support for intelligent research on low-temperature hot stamping.

The experiment–simulation fusion database construction paradigm proposed in this study is highly versatile and can be extended to dataset development in various manufacturing fields, including metal forming and heat treatment processes.

## Figures and Tables

**Table 1:** Chemical composition of the 22MnB5 steel.

**Element**	C	Si	Mn	P	S	Cr	Ti	B	Al
**Value (wt.%)**	0.21	0.23	1.15	0.008	0.03	0.15	0.02	0.0007	0.01

**Table 2:** Process parameter ranges and sample distribution.

Parameters	Symbols	Units	Ranges	Control Types	Sample Distribution Characteristics
Forming temperature	T	°C	300–600	Measured-value correction	Evenly distributed interval
Holding time	t	s	0–15	Predetermined value	Offset normal distribution
Stamping speed	v	mm/s	1–10	Predetermined value	Evenly distributed interval

**Table 3:** Thermophysical parameters of 22MnB5 steel.

Parameter	Fitting Formula	Unit
Specific Heat Capacity [11]	$c_{p}\left(T\right)=\left(460+0.32T^{0.8}\right)\times 10^{3}$	N·mm/(kg·K)
Thermal conductivity [12]	$\lambda \left(T\right)=\frac{-0.015T+36}{1000}$	W/(mm·K)
Linear expansion coefficient [13]	$\alpha \left(T\right)=(1.1+0.0006\frac{T}{100})\times 10^{-5}$	1/K
Convective heat transfer coefficient [14]	$h_{air}\left(T\right)=(20+0.01T)\times 10^{-3}$	N·mm/(s·mm^2^·K)
Interfacial heat transfer coefficient [9]	$h_{contact}=(1500-10T)\times 10^{-3}$	N·mm/(s·mm^2^·K)

**Table 4:** Selected result data.

No.	Forming Temperature (°C)	Holding Time (s)	Forming Speed (mm/s)	Springback Angle (°)	Vickers Hardness (HV)
E1	300	1.7	1.7	1.56	379.3
E2	306.2	9.5	8.1	1.25	392.5
E3	367.9	14.7	1.8	0.81	462.6
E4	422.2	7.8	4.2	0.57	532.8
E5	466.8	9.9	5.3	0.09	520.2
E6	495	11.1	4.8	0.20	523.1
E7	518.5	10.7	6.2	0.29	527.8
E8	531.2	12.6	4.7	0.17	523.4
E9	562	11.6	5.7	0.13	535
E10	600	14.3	6.9	−0.46	517.3

**Table 5:** Variable composition and selection criteria of the fusion dataset.

Category	Variable	Unit	Description
Input variable	Forming temperature	°C	Temperature of the blank sheet at the onset of forming
Input variable	Holding time	s	In-die holding and cooling time
Input variable	Stamping speed	mm/s	Upper-die loading speed
Output variable	Springback angle	°	Geometric recovery after unloading
Output variable	Hardness	HV	Post-forming mechanical property

## Data Availability

The original contributions presented in this study are included in the article. Further inquiries can be directed to the corresponding author.

## Abbreviations

The following abbreviations are used in this manuscript:

LTHS	Low-temperature hot stamping
FEM	Finite element model
